# Supplementary material for: Analysis of PPARγ Signaling Activity in Psoriasis
Source: Int J Mol Sci. 2021 Aug 10;22(16):8603. doi: 10.3390/ijms22168603 (PMC8395241; doi:10.3390/ijms22168603)
Supplement: Supplementary file 1 [file ijms-22-08603-s001.zip › Supplemental materials_Analysis of PPARg signaling activity in psoriasis/Pathway models/Models images and html files/PPARG inhibits phenotypes assotiated with psoriasis/189830.html]

T-cell activation


# Cell Process T-cell activation

|  |  |
| --- | --- |
| URN | urn:agi-gocellproc:0042110 |
| Total Entities | 3 |
| Connectivity | 3449 |
| Name | T-cell activation |
| ObjectType | Cell Process |

---

|  |  |
| --- | --- |
| ChildConcepts | gamma-delta T-cell activation |
|  | alpha-beta T-cell activation |
|  | T-cell gene rearrangement |

---

|  |  |
| --- | --- |
| Pathway | Complement Alternative Pathway |
|  | Cortisol in Resolving Inflammation |
|  | MHC2-Mediated Antigen Presentation |
|  | Endogenous Peptide Antigen Presentation |
|  | MHC1-Mediated Antigen Presentation |
|  | Plasmin Effects in Inflammation |
|  | Macrophage M1 Lineage |
|  | CD16/CD14 Proinflammatory Monocyte Activation |
|  | Histidine-Rich Glycoprotein (HRG) |
|  | HRH2/4 -> IL6/10 Production |
|  | Dendritic Cells Function in Atherosclerosis |
|  | Dendritic Cells Function in Psoriasis |
|  | Dendritic Cell Activation in Systemic Lupus Erythematosis |
|  | Neutrophil and Macrophage Function in Systemic Lupus Erythematosus |
|  | Macrophage Function in Diabetes Mellitus Type 1 |
|  | Conventional Dendritic Cell Role in Diabetes Mellitus Type 1 |
|  | Graves Ophthalmopathy |
|  | Enterocyte Mediated Mucosal Damage in Celiac Disease |
|  | Crohn's Disease |
|  | Paneth-Cell Function in Crohn's Disease |
|  | Macrophage Dysfunction |
|  | Genes with Susceptibility for Crohn's Disease and Ulcerative Colitis |
|  | Dendritic Cell Dysfunction in Crohn's disease |
|  | Dendritic Cell Function in Ulcerative Colitis |
|  | Diffuse Large-B-Cell Lymphoma |
|  | Primary Mediastinal B-cell Lymphoma (PMBL) Subtype |
|  | Hodgkin and Reed-Sternberg Cells in Hodgkin Lymphoma |
|  | Hodgkin Lymphoma |
|  | Basophil Activation in Asthma |
|  | Antigen-Presenting Cell Role in Asthma |
|  | Dendritic Cell Dysfunction in Psoriatic Arthritis |
|  | Synovial Fibroblast Activation in Psoriatic Arthritis |
|  | Synovial Fibroblast Activation by Citokines in Rheumatoid Arthritis |
|  | Toll-like Receptors (TLR) in Periodontitis |
|  | Diabetes Induced Periodontitis |
|  | PAPA Syndrome |
|  | TNF Receptor Associated Periodic Syndrome |
|  | Mevalonate Kinase Deficiency |
|  | Blau Syndrome |
|  | Cryopyrin (NLPR3) Associated Periodic Syndromes |
|  | Familial Cold Autoinflammatory Syndrome 2 |
|  | Familial Mediterranean Fever |
|  | Immune System Role in Otitis Media |
|  | T-Cell Cytotoxic Activation |
|  | Plasmin Role in Glomerulonephritis |
|  | Toll-like Receptors in Pyelonephritis |
|  | Tumor Infiltrating Macrophages in Cancer Progression and Immune Escape |
|  | MHC1 Causes Antigen Presentation Failure in Cancer Immune Escape |
|  | Myeloid Derived Suppressor Cells in Cancer Immune Escape |
|  | Effector T-cell Inactivation in Cancer Immune Escape |
|  | n |
|  | PPARG inhibits phenotypes assotiated with psoriasis |
|  | efects selected |
|  | Neighbors of T-cell activation |
|  | dsRNA induces innate immunity and INFs in alveolar cells |
|  | Dendritic Cell Activation in COVID19 |
|  | 3\_Autophagy dysfunction in Crohn's disease |
|  | Figure 4\_Diabetes mellitus Type 1: conventional dendritic cell |

---

|  |  |
| --- | --- |
| MedScan ID | 4142110 |

---

|  |  |
| --- | --- |
| Alias | stimulations of T-cell |
|  | T lymphocyte activation |
|  | pre-T lymphocyte induction |
|  | T lymphoblast co-stimulation |
|  | T lymphocyte stimulation |
|  | T-cell costimulation |
|  | induction of T-cell |
|  | T-cell induction |
|  | T lymphoblast costimulation |
|  | pre-T lymphocyte stimulation |
|  | activation of T lymphocyte |
|  | T cell co-stimulation |
|  | T lymphoblast induction |
|  | T lymphocyte costimulation |
|  | pre-T lymphocyte costimulation |
|  | T-cell activation |
|  | T lymphocyte co-stimulation |
|  | T-cell stimulations |
|  | stimulation of T-cell |
|  | T lymphocyte induction |
|  | activation of T-cell |
|  | T-cell activations |
|  | T-cell stimulation |
|  | T lymphoblast stimulation |

---

|  |  |
| --- | --- |
| GO ID | 0042110 |

---
